# Safety and Immunogenicity of Standard and Double Doses of Hepatitis B Vaccine in Children after Liver Transplantation: An Open-Label, Randomised Controlled Trial

**DOI:** 10.3390/vaccines10010092

**Published:** 2022-01-08

**Authors:** Palittiya Sintusek, Supranee Buranapraditkun, Piyaporn Wanawongsawad, Nawarat Posuwan, Pattarawat Thantiworasit, Nasamon Wanlapakorn, Jettanong Klaewsongkram, Narissara Suratannon, Nataruks Chaijitraruch, Voranush Chongsrisawat, Yong Poovorawan

**Affiliations:** 1 Division of Gastroenterology, Department of Pediatrics, King Chulalongkorn Memorial Hospital, Faculty of Medicine, Chulalongkorn University, Bangkok 10330, Thailand; Palittiya.S@chula.ac.th (P.S.); jsuttiruk@hotmail.com (N.C.); voranush.c@chula.ac.th (V.C.); 2 Thai Pediatric Gastroenterology, Hepatology and Immunology (TPGHAI) Research Unit, King Chulalongkorn Memorial Hospital, Faculty of Medicine, Chulalongkon University, The Thai Red Cross Society, Bangkok 10330, Thailand; bsuprane2001@yahoo.com; 3 Division of Allergy and Clinical Immunology, Department of Medicine, King Chulalongkorn Memorial Hospital, Faculty of Medicine, Chulalongkorn University, Thai Red Cross Society, Bangkok 10330, Thailand; pattarawatth6@gmail.com (P.T.); Jettanong@gmail.com (J.K.); 4 Center of Excellence in Vaccine Research and Development (Chula Vaccine Research Center-Chula VRC), Faculty of Medicine, Chulalongkorn University, Bangkok 10330, Thailand; 5 Excellence Center for Organ Transplantation, King Chulalongkorn Memorial Hospital, Bangkok 10330, Thailand; piyaporn.noonun@gmail.com; 6 Center of Excellence in Clinical Virology, Department of Pediatrics, Faculty of Medicine, Chulalongkorn University, Bangkok 10330, Thailand; nawarat.po@gmail.com (N.P.); patarakitnirun@hotmail.com (N.W.); 7 Pediatric Allergy and Clinical Immunology Research Unit, Division of Allergy and Immunolgy, Department of Pediatrics, King Chulalongkorn Memorial Hospital, Faculty of Medicine, Chulalongkorn University, The Thai Red Cross Society, Bangkok 10330, Thailand; mayzped@gmail.com

**Keywords:** children, hepatitis B virus, de novo hepatitis B, liver transplantation, vaccine, immunization

## Abstract

A high prevalence of hepatitis B (HepB) antibody loss after liver transplantation (LT) and de novo HepB infection (DNH) was documented, hence revaccination to prevent DNH is crucial. This study aimed to compare the safety and immunogenicity of two HepB vaccine regimens in liver-transplanted children. Liver-transplanted children who were previously immunised but showed HepB surface antibodies (anti-HBs) ≤ 100 mIU/mL were randomised to receive a standard three-dose (SD) and double three-dose (DD) vaccine intramuscularly in months 0–1–6. Anti-HBs and T-cell-specific response to the HepB antigen were assessed. A total of 61 children (54.1% male, aged 1.32 ± 1.02 years) completed the study without any serious adverse reaction. The seroprotective rate was 69.6% vs. 60% (*p* = 0.368) and 91.3% vs. 85% (*p* = 0.431) in SD and DD after the first and third 3-dose vaccinations, respectively. The geometric mean titre (95% confidence interval) of anti-HBs in SD and DD were 443.33 (200.75–979.07) vs. 446.17 (155.58–1279.50) mIU/mL, respectively, at completion. Numbers of interferon-γ-secreting cells were higher in hyporesponders/responders than in nonresponders (*p* = 0.003). The significant factors for the immunologic response to HepB vaccination were anti-HB levels prevaccination, tacrolimus trough levels, and time from LT to revaccination. SD and DD had comparative immunogenicity and were safe for liver-transplanted children who were previously immunised.

## 1. Introduction

Hepatitis B (HepB) infection poses a major public health challenge worldwide [1]. Approximately 12.4% of people infected with HepB have chronic infection, and 15–20% of chronic carriers die from cirrhosis or hepatocellular carcinoma [2]. Since the HepB universal vaccination programme for newborns started in the year 1992, the prevalence of HepB infection in many countries, including Thailand, has been rapidly decreasing due to the high efficacy of the vaccine [3]. Generally, the level of antibodies against the HepB surface antigen (anti-HBs) declines to less than the seroprotective level (<10 mIU/mL) in healthy adolescents and adults. However, a booster dose is not indicated if they had been primarily immunised, because the rapid anamnestic response could occur after HepB exposure [4]. Unlike healthy persons, disappearance of HB antibodies in liver-transplanted children might indicate loss of protection, as evidenced by de novo HepB infection (DNH) [5]. There has been no cut-off anti-HB level that is adequate for DNH prevention. However, the rapid decline of anti-HB levels in liver-transplanted children has led some countries such as China and Taiwan to adopt a higher reference level of anti-HBs >200 and >1000 mIU/mL, respectively, for booster vaccines [6,7].

DNH is defined as the development of new HepB surface antigen (HBsAg) positivity after LT in recipients who were previously negative for HBsAg. This condition was recognised in the 1990s [8,9] in recipients who received a HepB core antibody (HbcAb)-positive liver graft. DNH is a serious concern not because of long-term liver consequences, but due to the increased risk of acute graft rejection [10]. Consequently, a strategy to prevent DNH is crucial. Several studies have postulated that HepB vaccination prevents DNH in liver-transplanted children [6,7,11,12,13]. This practice is very cost-effective; however, some children must receive frequent booster doses during their hospital visit to maintain the high level of HepB antibodies [6].

Various strategies of HepB vaccination have been implemented to increase the immunogenicity in adults, including the use of multiple double doses, different types of vaccines and routes of administration, and adjuvants [14]. In HIV-infected adults, a recent meta-analysis showed that a double-dose HepB vaccination series could improve humoral immunogenicity for more than 1 year [15]. In liver-transplanted children, humoral and cellular responses after a booster dose are adequate only for a short term, and the best way to achieve and maintain long-term protection against HepB has been elusive so far. Therefore, this study aimed to evaluate the safety and immunogenicity of a double-dose compared to a standard-dose HepB vaccination series in liver-transplanted children with immunologic loss. Immunogenicity of the first dose as a “mock booster” and after a complete three-dose HepB vaccination of both regimens were also evaluated. Immunosuppressants targeting T cells might be a major factor of immunologic loss and poor response to vaccination. Therefore, we further aimed to study the function of T-cell-specific response to HepB vaccination, and determine the factors associated with an immunologic response after revaccination.

## 2. Materials and Methods

### 2.1. Study Design and Participants

This open-label, randomised clinical trial (RCT) was conducted between 2017 and 2020 at King Chulalongkorn Memorial Hospital in Thailand. The protocol was approved by the Institutional Review Board of Chulalongkorn University (IRB No.142/60) and was registered at the Thai Clinical Trials Registry (TCTR20180723002). Written informed consent was obtained from guardians and patients aged >12 years, and informed assent was obtained from patients aged 7–12 years at the time of enrolment, in accordance with the Declaration of Helsinki.

The eligibility criteria of liver-transplanted children recruited were as follows: (1) aged 1–18 years; (2) underwent LT for more than 6 months; (3) had previous HepB immunization; and (4) anti-HB titres less than 100 mIU/mL after LT. Liver-transplanted children were excluded if they were jaundiced (direct bilirubin >2 mg/dL), had a fever (body temperature >37.8 °C measured by nurses), or had cytomegalovirus or Epstein–Barr viremia within 7 days before the date of enrolment.

#### 2.1.1. Randomisation and Masking

The list of participants was initially ranked by the time since LT and then randomly allocated in blocks of 4 at a 1:1 ratio to receive either a standard 3-dose HepB vaccine (SD) (at a dose of 10 μg) or double 3-dose HepB vaccine (DD) (at a dose of 20 μg) according to a schedule of 0–1–6 months.

#### 2.1.2. Vaccine and Administration

The HepB vaccine used in this study was rDNA manufactured by GlaxoSmithKline, Belgium. According to the dosage and administration schedules of this rDNA vaccine, persons from birth through 19 years of age were intramuscularly administered with a dose of 0.5 mL (standard dose) at 0, 1, 6 months. Each 0.5 mL of vaccine contained 10 µg of HBsAg adsorbed to 0.25 mg of aluminium hydroxide. The vaccine lot numbers AHBVC680AD and AHBVC835AH were used for the SD group, and vaccine lot numbers AHBVC626AG and AHBVC871AH were used for the DD group.

#### 2.1.3. Study Procedures

Participants visited the hospital at 0–1–6 months for vaccination, and blood samples were collected for immunologic study before vaccination. The time of vaccination in each visit might have been delayed by −5 to 15 days from the targeted visits. The blood samples were continuously collected at 7–9 and 9–12 months according to the time of routine appointment by doctors in charge.

#### 2.1.4. Definition

Hyporesponders were participants who had anti-HBs <10 mIU/mL, but anti-HBs could rise to 10–99 mIU/mL after complete HepB vaccination.

Responders were participants who had anti-HBs >100 mIU/mL after complete HepB vaccination.

Nonresponders were participants who had anti-HBs <10 mIU/mL after complete HepB vaccination.

### 2.2. Outcomes

#### 2.2.1. Safety Assessments

Adverse events (AEs) of the vaccine (1 h after vaccination and 72 h via telephone call) were assessed and recorded by a research nurse. AEs were classified into local (pain, erythema, induration, edema, pruritus, hematoma, and inflammation.), systemic (fever, headache, fatigue, arthralgia, asthenia, diarrhoea, and nasopharyngitis), and serious (life-threatening illness, required hospitalisation, or significant disability).

#### 2.2.2. Immunogenicity Assessments

##### Humoral Response

HepB infection status was determined before vaccination by evaluating the levels of anti-HBs, HBsAg, and anti-HBc immunoglobulin M. Anti-HBs were measured at 0–1–6 months, 7–9, and 9–12 months by an automated enzyme-linked immunosorbent assay performed using the ARCHITECT system (Abbott, Wiesbaden, Germany) according to the manufacturer’s instructions, with a cut-off point of >1 mIU/mL [3].

##### Cellular Response

Peripheral blood mononuclear cells (PBMCs) were isolated and stored in liquid nitrogen until use. For cryopreservation, cells were resuspended in a freezing medium (FBS containing 10% dimethyl sulfoxide) at a concentration of 1 × 107 cells/mL.

To quantify the number of interferon-gamma (IFN-γ)-secreting T cells, HBV-specific T-cell responses were evaluated in PBMCs using a IFN-γ enzyme-linked immunospot (ELISpot). All assays were performed in duplicate. Briefly, 96-well nitrocellulose membrane plates (MAIPS45; Millipore, Bedford, MA, USA) were coated with 5 μg/mL antihuman IFN-γ (1-D1K) monoclonal antibodies (mAb) (Mabtech, Stockholm, Sweden) overnight at 4 °C. Then, the plates were washed and blocked with culture medium (RPMI 1640 with 10% FBS) for 1 h at room temperature (RT). PBMCs were cultured at a cell density of 250,000 cells/100 μL/well with HBsAg adr subtype recombinant protein (MyBioSource, USA) at a concentration of 5 μg/mL at 37 °C with 5% CO_2_ for 40 h. Culture medium alone served as a negative control, and phytohemagglutinin as a positive control. After incubation, the plates were washed with phosphate-buffered saline (PBS), and cells were incubated with 1 µg/mL antihuman IFN-γ-biotinylated mAb (7-B6-1 biotin; Mabtech, Stockholm, Sweden) in PBS for 3 h at RT. After washing with PBS, 100 μL streptavidin-ALP in PBS (1:1000 dilution) was added to each well, and the plates were incubated for 1 h at RT. After washing, 100 μL of the substrate solution (5-bromo-4-chloro-3-indolyl-phosphate/nitro blue tetrazolium) was added. The spots were developed until distinct spots emerged. The reaction was stopped by washing the membranes extensively with tap water and rinsing the underside of the membrane. The plates were air-dried. The spots were analysed using an ELISpot reader (Carl Zeiss, Jena, Germany). The mean numbers of IFN-γ-producing and spot-forming cells (SFCs) were calculated from duplicate assays. HBsAg-specific responses were calculated by subtracting background spots from the negative wells, and were expressed as SFC/106 PBMCs.

### 2.3. Statistical Analysis

The sample size was calculated for the RCT with binary outcomes under the assumption that children in the SD and DD groups would have anti-HBs >10 mIU/mL after vaccination of 30% individuals in the SD group and 70% individuals in the DD group, respectively, with alpha 0.05, and 80% power of test. Using two independent proportions without a continuity correction formula, the ratio of participants in both groups was found to be 1:1. At least 60 children were required for the study (30 participants in each arm). When 10% of dropouts were accounted for, a total of 68 participants were recruited in the present study.

Data analyses were performed using Statistical Package for the Social Sciences version 24.0.0 (SPSS Inc., Chicago, IL, USA) and Stata version 15.1(Stata Corp, LLC, College Station, TX, USA). Continuous and categorical data were presented as the mean ± standard deviation or median (interquartile range) and proportion or percentage as appropriate. The Mann–Whitney U test and unpaired t-test were used to compare continuous data as appropriate. The Fisher’s exact test and Chi-square test were used to compare discrete data as appropriate. Univariate analysis was performed for the independent factors of anti-HB seroconversion after revaccination. Statistical significance was set at *p* < 0.05. The geometric mean titre (GMT) was calculated from an anti-HB titre >1 mIU/mL and represented logarithmically. The study was statistically reviewed by a biomedical statistician at the Department of Statistics Science, Kasetsart University, Thailand.

## 3. Results

### 3.1. Study Populations

**Figure 1:**
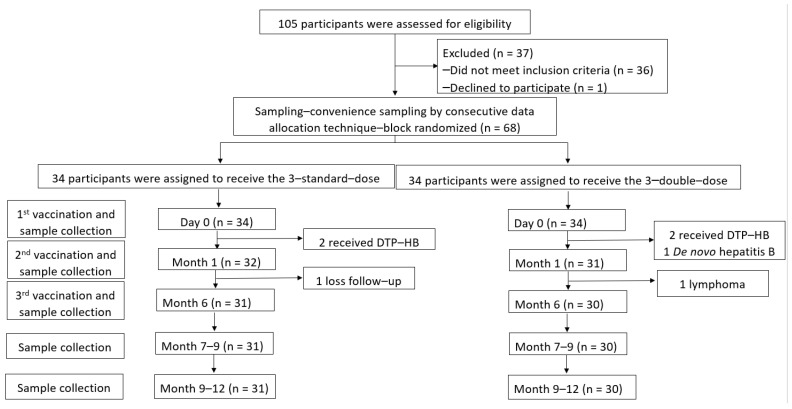
Enrollment, randomisation, and follow-up of participants.

A total of 105 children who underwent LT between 2003 and 2019 were assessed, and 68 participants aged 1.83 ± 1.23 years (47.5% male) were enrolled. There were 34 participants for each arm. However, anti-HB levels ≥100 mIU/mL were detected in two participants each in the SD and DD groups at baseline (before the vaccine was administered) due to combined diphtheria–tetanus–pertussis–HepB (DTP-HepB) vaccine administration by their local hospitals. One participant in the DD group was diagnosed with DNH after enrolment. After vaccination, one participant in the SD group could not visit the hospital at the proper time, and one participant in the DD group was diagnosed with post-transplant lymphoproliferative disorders that progressed to B-cell lymphoma. Therefore, 61 participants received the three-dose vaccine regimen according to the study protocol (31 and 30 children in the SD and DD groups, respectively) (Figure 1).

The ages were 1.06 (0.84, 2.87) (44.2% male) and 1.72 (1.19, 5.74) (52.9% male) years in SD and DD, respectively. Most of them received 1–2 immunosuppressants (83.8%), mainly tacrolimus (48.6%). The time from liver transplantation to HepB revaccination were 1.44 (0.66, 3.59) and 1.69 (0.67, 4.97) years, with anti-HB levels prior to HepB revaccination of 1.75 (0.40, 13.8) and 2.7 (0.8, 14) mIU/mL in SD and DD, respectively. These baseline demographic data and patient characteristics, including immunosuppressant trough levels and basic laboratory results between the SD and DD groups at the time of enrolment, did not show a statistically significant difference (Table 1).

### 3.2. Safety of HepB Revaccination

A total of 17 cases (0.9%) of AEs were recorded in a total of 198 doses of vaccine administered (12 and 5 cases of AEs in the SD and DD groups, respectively; *p* = 0.062). Pain at the injection site was the most common AE reported (n = 10), but it subsided within 72 h without any medication. Other AEs included itching at the injection site (n = 2), fever within 72 h after injection (n = 4), and diarrhoea (n = 1). None of the participants had transaminitis or graft rejection within two weeks of vaccination.

### 3.3. Humoral Response after HepB Revaccination

**Figure 2:**
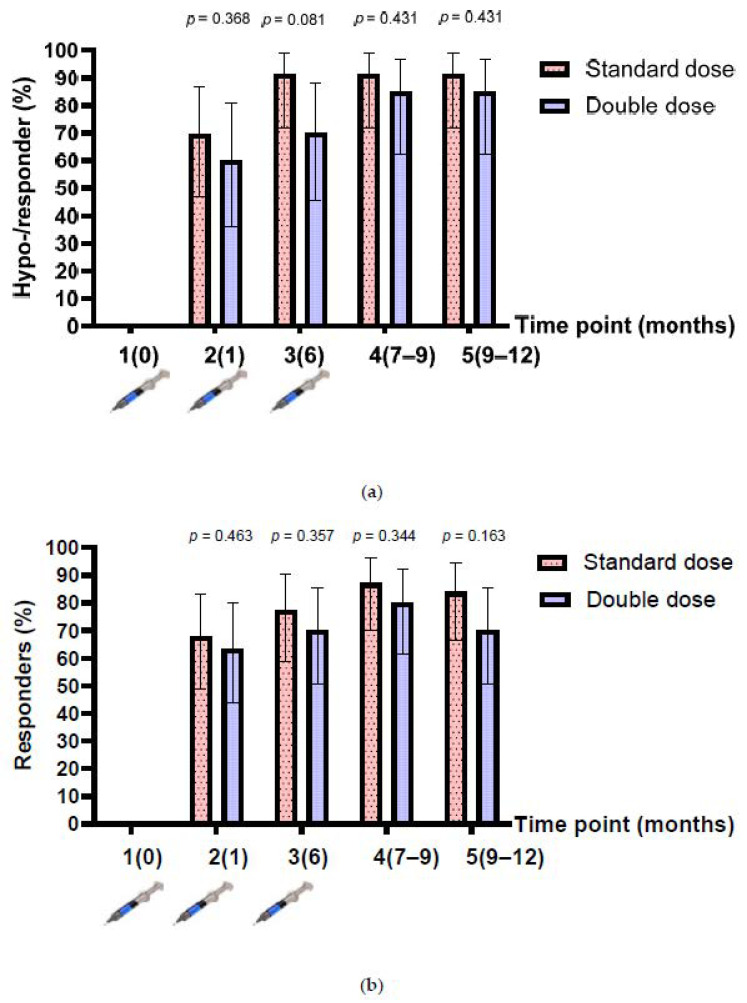
Anti-HBs response before and after 3-dose HepB vaccination. (**a**) Hyporesponders/responders from participants who had a baseline anti-HBs level of <10 mIU/mL (n = 43); (**b**) responders from participants who had a baseline anti-HBs level of <100 mIU/mL, before and after 3-dose HepB vaccination (n = 61).

The baseline anti-HBs level after enrolment was 2.1 (IQR 0.5, 13.9) mIU/mL, and 18 participants had anti-HBs levels between 10 and 99 mIU/mL (8 and 10 participants in the single- and double-dose arms, respectively). Therefore, the number of hyporesponders (anti-HBs between 10 and 99 mIU/mL after HepB vaccination) were assessed in 43 participants, while the responders (anti-HBs >100 mIU/mL) were assessed in 61 participants. The rates of hyporesponders/responders after the one-dose and three-dose vaccinations were 69.6% (95% confidence interval (CI), 47.1–86.8%) vs. 91.3% (95% CI, 72–98.9%) (*p* = 0.067) in the SD group; and 60% (95% CI, 36.1–80.9%) vs. 85.0% (95% CI, 62.1–96.8%) (*p* = 0.078) in the DD group (n = 43). The rates of responders after the one-dose and three-dose vaccinations were 67.7% (95% CI, 48.6–83.3%) vs. 87.1% (95% CI, 70.2–96.4%) (*p* = 0.064) in the SD group; and 46.3% (95% CI, 43.9–80.1%) vs. 80.0% (95% CI, 61.4–92.3%) (*p* = 0.126) in the DD group (n = 61). Overall, the proportion of hyporesponders/responders in the SD group was higher than that in the DD group at all time points, but the difference was not statistically significant (Figure 2a,b).

**Figure 3:**
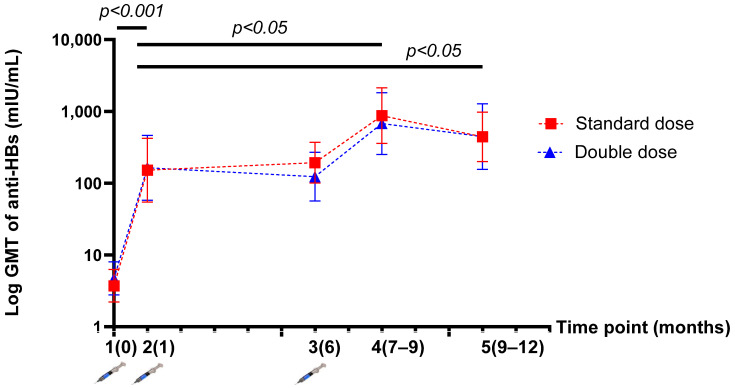
Demonstration of the log geometric mean titre (GMT) of anti-HBs levels before and after vaccination at five time points (per protocol population).

The GMT of anti-HBs was not significantly different between the SD and DD groups at five time points. The GMT of anti-HBs levels after the one-dose and three-dose vaccinations were 151.93 (95% CI, 54.67–422.26) vs. 871.65 (95% CI, 358.42–2119.78) mIU/mL (*p* = 0.032) in the SD group; and 163.75 (95%CI, 57.74–464.38) vs. 677.18 (95%CI, 251.80–2401.82) mIU/mL (*p* = 0.043) in the DD group (Table 2). After HepB revaccination, there was a significantly higher level of anti-HBs than at the beginning of the study in both groups (*p* < 0.001). Moreover, the GMT of anti-HBs levels after the three-dose vaccination was significantly higher than that after the one-dose vaccination or “mock booster” (*p* < 0.05) in both groups (Figure 3).

### 3.4. Cellular Response after HepB Vaccination

T-cell-specific response to the HepB antigen was assessed using an IFN-γ ELISpot assay. Blood samples were collected from 42 participants (21 participants from each group) at three time points, from which adequate PBMCs were extracted for this and future studies.

**Figure 4:**
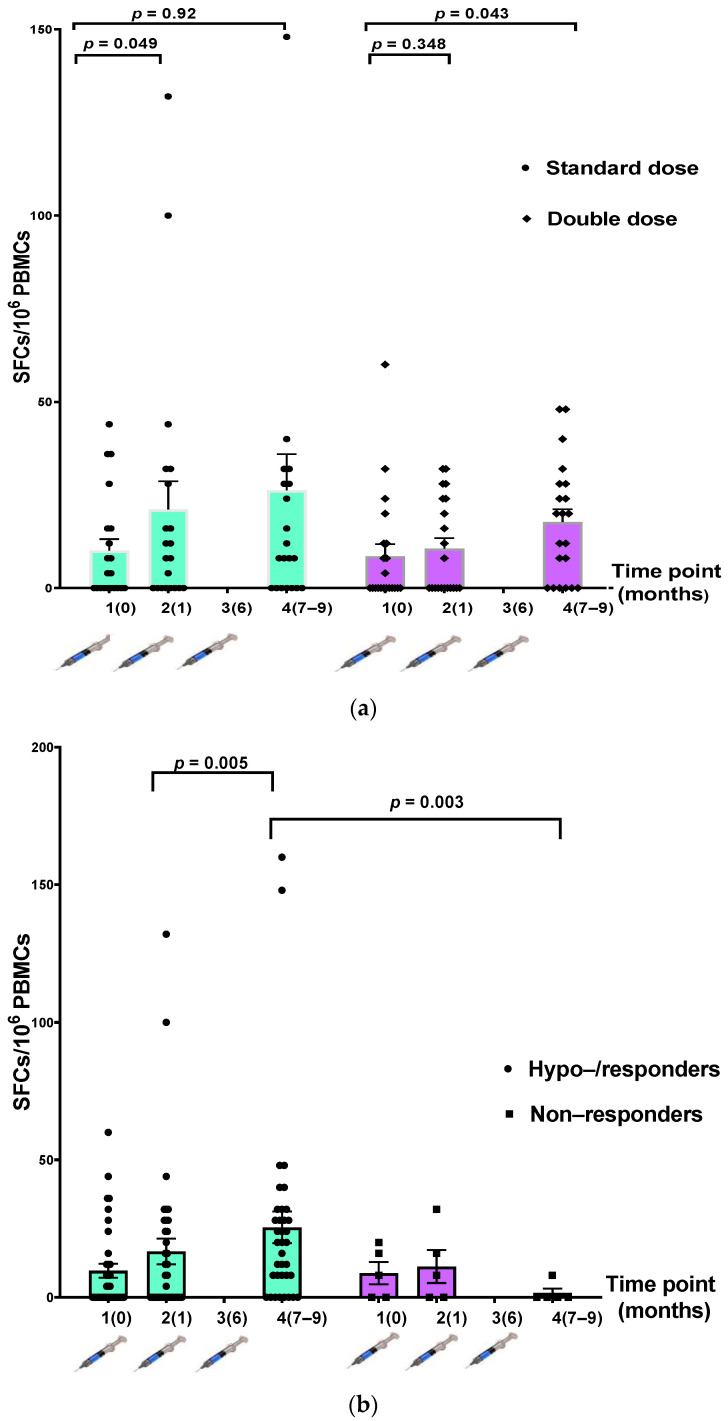
Comparison of T-cell-specific response to hepatitis B antigen measured using IFN-γ ELISpot assay at three time points. (**a**) The standard-dose (SD) (n = 21) and double-dose (DD) (n = 21) groups; (**b**) hyporesponders/responders (n = 37) and nonresponders (n = 5).

Comparing the IFN-γ-secreting cells between the participants in the SD and DD groups, we found no statistically significant increase in the number of IFN-γ-secreting cells after vaccination at each time point (Table 3, Figure 4a). When participants were divided into hyporesponders/responders (n = 37) and nonresponders (n = 5), there was a significantly higher number of IFN-γ-secreting cells after the three-dose vaccination in the responder group (*p* = 0.003) (Table 3, Figure 4b).

### 3.5. Factors Related to Humoral Immune Response after Three-Dose Vaccination

Baseline characteristics of hyporesponders/responders and nonresponders were compared to determine the factors associated with immunologic response to HepB vaccination (Table 4). Factors significantly associated with no seroprotective antibody levels (anti-HBs <10 mIU/mL) after HepB vaccination included early time of revaccination (*p* = 0.03), lower anti-HBs levels prevaccination (*p* < 0.001), and higher tacrolimus trough levels (*p* = 0.028).

## 4. Discussion

This was an RCT comparing the efficacy of an SD with a DD of HepB vaccination in liver-transplanted children. There was a statistically insignificant difference in the response rate and AEs in participants after SD vaccination compared to those after DD vaccination. The response rate and the GMT of anti-HBs titres were higher after the third dose compared to the first dose (mimicking a scenario of a booster dose) in both groups. Moreover, the high response rate and the GMT of anti-HBs titres after a three-dose vaccination were observed during a short-term follow-up (392 (IQR 306, 448) days from enrolment). Time from LT to revaccination, anti-HBs levels at baseline, and tacrolimus trough levels were the factors significantly associated with a robust immunologic response to the HepB revaccination.

Booster doses of HepB vaccine in immunocompetent individuals with loss of seroprotection despite previous immunisation are not recommended [4]. HBsAg-specific immune memory can have an anamnestic response, normally at 5–8 days after re-exposure to HBsAg, and peaking after about 14 days [16,17]. However, there is little information on HBsAg-specific immune memory and the response after HBsAg exposure in liver-transplanted recipients. The presence of anti-HBs is the simplest way to demonstrate durable protection. As anti-HB levels rapidly decline in these vulnerable children [5,18], maintaining a higher anti-HB level to guarantee protection is reasonable. A recent cohort study by Song et al. showed that maintaining anti-HB levels >200 mIU/mL could effectively prevent DNH [7], while Lin et al. suggested the cut-off for anti-HBs levels was >1000 mIU/mL instead [6]. To avoid repeated vaccination with booster doses, this was the first RCT study to compare the safety and immunogenicity of two vaccine regimens (SD and DD) for HepB reimmunisation in liver-transplanted children. While there were no data on other HepB vaccine regimens in liver-transplanted children, several HepB vaccine regimens have been assessed in HIV-infected people. Several studies found a high response rate and a greater GMT of anti-HBs after 72 weeks of follow-up in multiple doses of the double-dose regimen [19,20,21,22,23,24]. Regarding these results, the international guidelines recommend a three double-dose rescue HepB vaccination in HIV-infected populations with anti-HB loss [18,25,26]. Similarly, the current study demonstrated that a three-dose regimen of the HepB vaccine induced a higher response rate and higher anti-HB levels than after the first dose. However, the efficacy of DD was not superior to that of the SD regimen after short-term follow-up, which was not consistent with the results of studies in HIV-infected populations. The HIV-infected population might have had more T-cell defects that were not in conjunction with liver-transplanted children, who received mainly T-cell immunosuppressive agents, so that the dosage of these immunosuppressants could be gradually decreased after 6–12 months post-transplant. Other possible contributing factors that might be considered include age, specific HLA-DPB1 gene, follicular T cells, and activated antibody-secreting cells (ASC) [27,28]. Hence, further studies with a larger sample size and stratified according to the described possible contributing factors are merited, with the need of modifying the dose of HepB vaccine in liver-transplanted children. Furthermore, DD might be beneficial for liver-transplanted children who did not respond well to the SD regimen. Study of the DD regimen in these selected cases in parallel with assessment of immunogenicity after revaccination is needed in our ongoing research.

HBsAg-specific T cells might play a major role in the humoral response after revaccination. Consequently, we conducted an ELISpot assay to evaluate the cellular immune response to the HepB vaccine, focusing on T-helper1-cell response, and found a significantly higher than the baseline number of IFN-γ-secreting cells in responders than in hyporesponders. The results of the current study were not in line with the study by Carollo et al. [29], but were similar to the study by Ni et al. [11], which showed a higher level of surrogate markers for both T-helper1 and T-helper2 cells after HepB revaccination. Regarding other types of cellular immune responses, Bauer et al. [30] showed that the specific induction of regulatory T cells (Tregs) could contribute to the poor humoral response after HepB revaccination in LT recipients, similar to predominant Tregs inducing immune tolerance in HepB-infected individuals with persistent HepB infection. Regarding B-cell function in HepB revaccination, Bolther et al. [31] studied both regulatory B (Breg) cells and IFN-γ-positive T cells to predict humoral response in healthy volunteers. They found a robust Breg or T-helper1-cell response that did not correlate with anti-HB loss in these immunocompetent individuals. To date, there have been no studies on the role of specific B cells (transitional, mature naïve, IgM memory, and switched-memory B cells) in the immune response. Moreover, other cells, such as Tregs, T follicular helper cells, and long-lived plasma cells, might be involved in the humoral cell response after revaccination in liver-transplanted recipients.

The current study found that 8.2% of participants failed to reach seroprotective antibody levels (anti-HBs > 10 mIU/mL) after a three-dose HepB vaccination regime, which was attributed to significantly associated factors, including early time from LT to vaccination, high tacrolimus trough levels, and a very low anti-HBs level at the time of revaccination. In theory, the effect of high exposure to immunosuppressants at the early stage after LT could explain the poor immune response to vaccines [32]. Furthermore, the low anti-HBs level at the time of revaccination might reflect weak cellular immunity at baseline. Therefore, strategies to improve the immunologic response after revaccination in liver-transplanted children include the administration of a booster dose of HepB vaccine before LT to maintain high levels of anti-HBs and balance the time for revaccination after LT, which should not be earlier than 3 months post-LT when the dose of immunosuppressants administered is high. Moreover, long-term monitoring of patients should be carried out, and factors that could maintain the longevity of anti-HBs levels should be evaluated.

There were several strengths to this study. First, the well-designed RCT compared the potential vaccination regimens in their abilities to effectively trigger an enhanced immune response after revaccination in children who underwent LT. Second, this study evaluated both cellular and humoral immunogenicity of the HepB vaccine. However, there were some limitations to this study. Other cell types, such as Treg, memory B cells, follicular T cells, ASCs, etc., and genetic predisposition might be factors of the poor immune response after revaccination, which were not investigated in this study.

## 5. Conclusions

A rebooster dose of HepB vaccine to keep high anti-HB levels in liver-transplanted children is crucial. A standard three-dose or double three-dose HepB vaccine according to a schedule of 0–1–6 months for liver-transplanted children who had anti-HB loss had comparative immunogenicity and was safe. For a robust immunologic response to HepB revaccination, time from LT to revaccination, anti-HB levels at baseline, and tacrolimus trough levels were the significant factors to be considered prior to reimmunization. The present study suggested that HepB revaccination should be introduced earlier but after 3 months post-LT, when anti-HB levels are not less than 10 mIU/mL.

## Figures and Tables

**Table 1:** Baseline patient characteristics before hepatitis B revaccination.

Parameters	Standard Dose (n = 34)	Double Dose (n = 34)
Age at transplantation (years)	1.06 (0.84, 2.87)	1.72 (1.19, 5.74)
Male (n, % male)	15 (44.2)	18 (52.9)
Time at hepatitis B revaccination (years)	1.44 (0.66, 3.59)	1.69 (0.67, 4.97)
Number of immunosuppressants administered (n, %)		
None	1 (2.9)	1 (2.9)
One	15 (44.1)	15 (44.1)
Two	13 (38.2)	14 (41.2)
Three	5 (14.7)	4 (11.7)
Type of immunosuppressants administered (n, %) prevaccination		
Tacrolimus	15 (44.1)	18 (52.9)
Cyclosporin	5 (14.7)	4 (11.8)
More than one	14 (40.2)	12 (35.2)
Level of immunosuppression (ng/mL)		
Tacrolimus	3.4 (2.7, 5.4)	3.7 (3.0, 4.8)
Cyclosporin	217 (123, 629)	386 (106, 937.5)
Pretransplant disease; BA (n, %)	26 (76.5)	24 (70.5)
Complications (PTLD, graft rejection, and surgical conditions) (n, %)	18 (52.9)	14 (41.2)
Anti-HBs level (mIU/mL) prevaccination	1.75 (0.40, 13.8)	2.7 (0.8, 14)
Anti-HBs level prevaccination (n, %)		
<10 mIU/mL	24 (70.6)	22 (64.7)
10–99 mIU/mL	8 (23.5)	10 (29.4)
≥100 mIU/mL	2 (5.9)	2 (5.9)
Laboratory investigation		
SGOT (IU/L)	41 (35.5, 51)	41 (32, 55)
SGPT (IU/L)	31 (19, 42)	26 (18, 49.5)
GGT (IU/L)	24 (18, 63)	34 (22, 83.5)
Albumin (g/dL)	4.2 (4, 4.4)	4.1 (3.85, 4.2)
Hb (g/dL)	12 (10.8, 12.7)	10.8 (9.9, 12.2)
WBC (106/L)	8290 (6840, 12,150)	7760 (5605, 9835)
Lymphocyte count (×106/L)	1365 (288, 6180)	2900 (660, 4480)
Platelet count (×109)	244 (203.5, 313)	230 (178.5, 320)

Data are median (IQR) or n (%). PTLD, post-transplant lymphoproliferative disease; SGOT, serum glutamic oxaloacetic transaminase; SGPT, serum glutamic pyruvic transaminase; GGT, gamma-glutamyltransferase; Hb, haemoglobin; WBC, white blood cell.

**Table 2:** Demonstration of the geometric mean titre (GMT) of anti-HBs levels before and after vaccination at five time points (per protocol population).

Table	Time from Enrolment (Days)	GMT (95% CI), mIU/mL		*p*-Value
		Standard Dose (n = 31)	Double Dose (n = 30)	
1	0	3.71 (2.20–6.25)	4.70 (2.76–7.99)	0.498
2	28 (28, 35)	151.93 (54.67–422.26) **	163.75 (57.74–464.38) **	0.160
3	193 (175, 210)	193.58 (100.79–371.79)	123.39 (56.52–269.34)	0.124
4	280 (262, 329)	871.65 (358.42–2119.78) *	677.18 (251.80–2401.82) *	0.207
5	392 (306, 448)	443.33 (200.75–979.07) *	446.17 (155.58–1279.50) *	0.164

** *p* < 0.001 at time point 1, * *p* < 0.05 at time point 2. GMT, geometric mean titre.

**Table 3:** Comparison of T-cell-specific response to hepatitis B antigen measured using IFN-γ ELSIPOT assay at three time points.

Time Point	IFN-*γ*-Secreting Cells (SFCs/10^6^ PBMCs)		*p*-Value	IFN-*γ*-Secreting Cells (SFCs/10^6^ PBMCs)		*p*-Value
	Standard Dose (n = 21)	Double Dose (n = 21)		Hypo-/Responders (n = 37)	Nonresponders (n = 5)	
1 (first dose)	4 (0, 16)	0 (0, 12)	0.559	0 (0, 12)	8 (0, 12)	0.690
2 (second dose)	8 (0, 28) *	0 (0, 24)	0.420	8 (0, 24)	0 (0, 12)	0.624
3 (third dose)	-	-	-	-	-	-
4 (after third dose)	8 (0, 28)	20 (0, 28) *	0.778	20 (8, 28) *	0 (0, 0)	0.003

SFCs, spot-forming cells; PBMCs, peripheral blood mononuclear cells. * *p* < 0.05 at time point 1. Data are median (IQR).

**Table 4:** The characteristics and laboratory data between hyporesponders/responders and nonresponders.

Parameters	Hyporesponders/ Responders (n = 38)	Nonresponders (n = 5)
Age at transplantation (years)	1.35 (0.83, 3.01)	3.45 (0.71, 13.58)
Male (n, % male)	20 (52.6)	2 (50)
Time at hepatitis B revaccination (years)	1.95 (0.66, 4.95)	0.58 (0.54, 0.65)
Vaccination protocol, standard dose (n, %)	21 (55.3%)	2 (40%)
Number of immunosuppressants administered (n, %)		
None	1 (2.4)	-
One	30 (50)	5
Two	6 (38.1)	-
Type of immunosuppressants administered (n, %)		
Tacrolimus	30 (78.6)	5 (93.3)
Cyclosporin	6 (19)	-
None	1 (2.4)	-
Level of immunosuppression (ng/mL) prevaccination		
Tacrolimus	3.6 (2.6, 5.7)	6.7 (5.8–7.8)
Cyclosporin	209 (94, 941)	-
Pretransplant diseases (n, %)		
BA	28 (73.8)	4 (80)
Others	9 (26.2)	1 (20)
Complications (PTLD, rejection, and surgical conditions) (n, %)	20 (52.4)	1 (40)
Anti-HBs level (mIU/mL) prevaccination	5.5 (0.6, 13.9)	1 (0.2, 1.7)
Laboratory investigation		
SGOT (IU/L)	41 (33, 54)	42 (28, 50)
SGPT (IU/L)	30 (19, 46)	28 (18, 56)
GGT (IU/L)	30 (21, 66)	113 (28, 181)
Albumin (g/dL)	4.1 (3.9, 4.3)	3.9 (3.6, 4.4)
Hb (g/dL)	11.8 (10.4, 12.7)	10.2 (8.8, 14.2)
WBC (×106/L)	7810 (6332, 10,862)	8675 (3642, 9627)
Lymphocyte count (×106/L)	619 (266, 3808)	4020 (941, 5527)
Platelet count (×109)	232 (174, 313)	298 (204, 425)

Data are median (IQR) or n (%). PTLD, post-transplant lymphoproliferative disease; SGOT, serum glutamic oxaloacetic transaminase; SGPT, serum glutamic pyruvic transaminase; GGT, gamma-glutamyltransferase; Hb, haemoglobin; WBC, white blood cell.

## Data Availability

Not applicable.
